# Targeting signaling pathways with andrographolide in cancer therapy (Review)

**DOI:** 10.3892/mco.2024.2779

**Published:** 2024-09-05

**Authors:** Nur Shahirah Shaharudin, Gurmeet Kaur Surindar Singh, Teh Lay Kek, Sadia Sultan

**Affiliations:** 1 Department of Pharmaceutical Chemistry, Faculty of Pharmacy, Universiti Teknologi MARA, Puncak Alam, Selangor 42300, Malaysia; 2 Department of Pharmacology and Life Sciences, Faculty of Pharmacy, Universiti Teknologi MARA, Puncak Alam, Selangor 42300, Malaysia; 3 Faculty of Pharmacy, Brain Degeneration and Therapeutics Research Center, Universiti Teknologi MARA, Shah Alam, Selangor 40450, Malaysia; 4 Faculty of Pharmacy, Biotransformation Research Center, Universiti Teknologi MARA, Shah Alam, Selangor 40450, Malaysia

**Keywords:** andrographolide, anticancer, NF-κB pathway, HIF-1 pathway, JAK/STAT pathway, PI3K/AKT/mTOR pathway, Wnt/β-catenin pathway, MAPK pathway

## Abstract

Terpenoids are a large group of naturally occurring organic compounds with a wide range of components. A phytoconstituent in this group, andrographolide, which is derived from a plant called *Andrographis paniculate*, offers a number of advantages, including anti-inflammatory, anticancer, anti-angiogenesis and antioxidant effects. The present review elucidates the capacity of andrographolide to inhibit signaling pathways, namely the nuclear factor-κB (NF-κB), hypoxia-inducible factor 1 (HIF-1), the Janus kinase (JAK)/signal transducer and activator of transcription (STAT), phosphatidylinositol-3-kinase (PI3K)/AKT/mammalian target of rapamycin (mTOR), Wnt/β-catenin and mitogen-activated protein kinase (MAPK) pathways, which are involved in cellular processes and responses such as the inflammatory response, apoptosis and angiogenesis. Inhibiting pathways enables andrographolide to exhibit its anticancer effects against breast, colorectal and lung cancer. The present review focuses on the anticancer effects of andrographolide, specifically in breast, colorectal and lung cancer through the NF-κB, HIF-1 and JAK/STAT signaling pathways. Therefore, the Google Scholar, PubMed and ScienceDirect databases were used to search for references to these prevalent types of cancer and the anticancer mechanisms of andrographolide associated with them. The following key words were used: Andrographolide, anticancer, JAK/STAT, HIF-1, NF-κB, PI3K/AKT/mTOR, Wnt/β-catenin and MAPK pathways, and the literature was limited to studies published between 2010 to 2023. The present review article provides details about the different involvements of signaling pathways in the anticancer mechanisms of andrographolide.

## 1. Introduction

Given its constant state of evolution and adaptation, modern medicine is keeping pace with current trends by integrating herbal extracts that have long been traditionally utilized as complementary components to medical products. The difference between herbal extracts and pharmaceutical preparations is that the former contains more than one active chemical ingredient, while the latter usually contains only one such ingredient that demonstrates clear pharmacological activities (1). However, the composition of chemical extracts depends on the geographical distribution of the herbs (2). In addition, active pharmacophores may be identified in herbal extracts, such as curcumin, which is found in the curcuma root (3); epigallocatechin, which is derived from green tea (3,4); and resveratrol, which is obtained from red wine (3).

**Figure 1:**
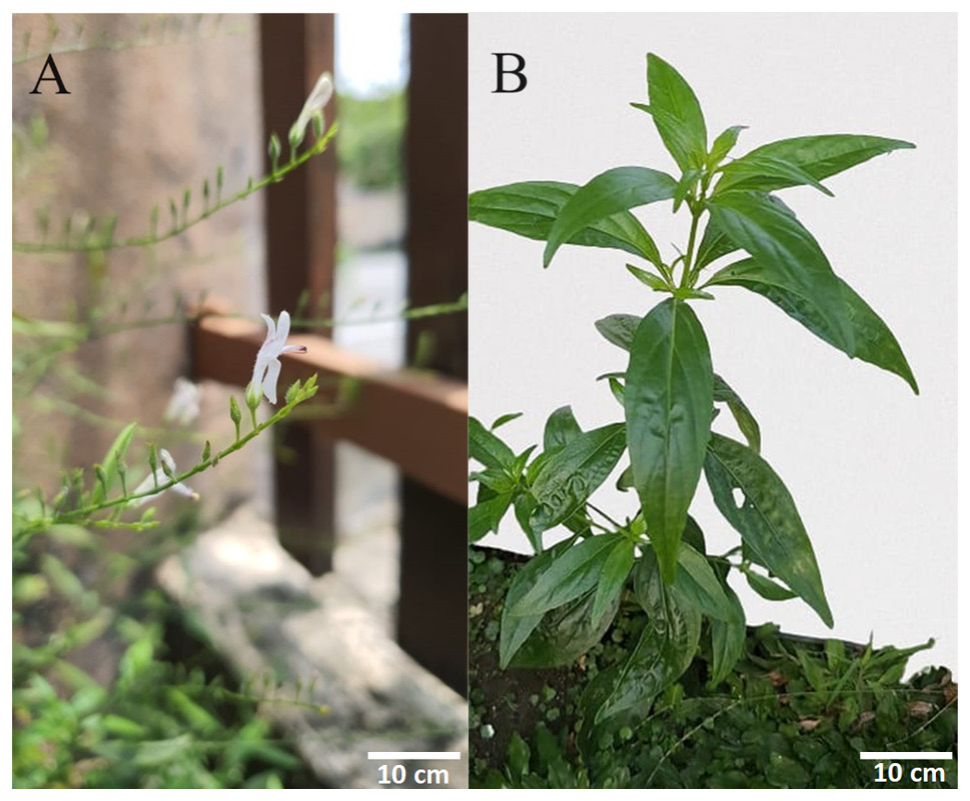
*Andrographis paniculata* plant. (A) An image of the flower and (B) the aerial part of the plant, consisting of the leaves and stem.

**Figure 2:**
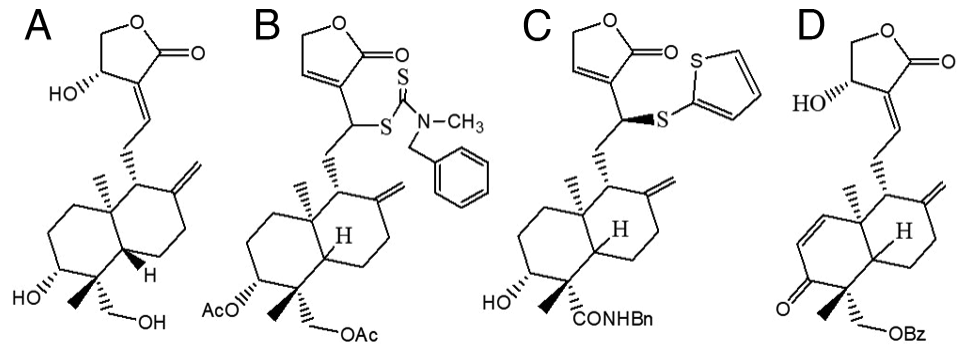
Chemical structures of andrographolide and its derivatives. Chemical structures of (A) andrographolide (19), (B) 12-dithiocarbamate-14-deoxyandrographolide analogue (16), (C) 3,19-analogue of 12-thioether andrographolide (17) and (D) 19-(2-furoyl)-1,2-didehydro-3-ox-andrographolide (15). Structures were drawn using ChemDraw Ultra^®^ (version 7.0.1; Revvity Signals Software, Inc.).

Natural compounds have been gaining considerable attention in the pharmaceutical industry with regard to the prevention and treatment of cancer. Several natural compounds have been reported to have anticancer effects, such as curcumin, quercetin, matrine and andrographolide (5). Derived from the plant *Andrographis paniculata*, the andrographolide compound offers numerous advantages, particularly as a herbal medicine, which is favored in Asian countries such as India and China. As a herbal medicine, andrographolide has several advantages, including being used as a therapy for common illnesses and infections such as colds, tonsillitis, diarrhea, wounds and ulcers. Meanwhile, the plant broth can be utilized to treat jaundice, dermatitis, and liver diseases (6). Varying quantities of this compound can be acquired from distinct plant components and different geographical locations. Plants growing in locations with moderate temperatures (25-28˚C) have a higher andrographolide concentration compared with plants in locations with temperatures <25˚C or >30˚C (6). Andrographolide can be obtained both naturally and synthetically. Naturally, the compound can be extracted from the plant, *Andrographis paniculata*, through a process of extraction, isolation and purification to obtain the pure andrographolide compound (7). The concentration of andrographolide extracted from a plant ranges between 0.054-4.686%, with the leaves containing the highest concentration compared with the stem of the plant, flowering tops and roots (8). The leaves and flowers of the *Andrographis paniculata* plant are shown in Fig. 1. Andrographolide belongs to the class of terpenes, namely diterpenes (9). The molecular formula for andrographolide is C_20_H_30_O_5_, and its IUPAC name reported on PubChem is (3*E*,4*S*)-3-[2-[(1*R*,4*aS*,5*R*,6*R*,8*aS*)-6-hydroxy-5-(hydroxymethyl)-5,8*a*-dimethyl-2-methylidene-3,4,4*a*,6,7,8-hexahydro-1*H*-naphthalen-1-yl]ethylidene]-4-hydroxyoxolan-2-one (Fig. 2A) (10-12).

Andrographolide has attracted interest due to its extensive range of pharmacological effects, which include anticancer, antimalarial, anti-inflammatory, antidiabetic, antiviral and antibacterial benefits (13). Andrographolide and its derivatives may be able to prevent and treat cancer, as well as exhibit anticancer effects through the inhibition of the growth, multiplication and metastasis of numerous types of cancer cells, such as breast, lung, colorectal, bladder and colon cancer cells as well as prostate carcinoma and leukemic cells (14). A potent compound, 19-(2-furoyl)-1,2-didehydro-3-ox-andrographolide (Fig. 2D), can inhibit the proliferation of breast cancer cell lines, especially HCT116 and MCF-7, with an IC_50_ <8 for both cell lines (15). Furthermore, the potent andrographolide derivative, 12-dithiocarbamate-14-deoxyandrographolide analogue (Fig. 2B), has an anticancer effect on nine different types of cancer cells, especially MCF-7 and KKU-055, which are human breast cancer cells and human cholangiocarcinoma cells, respectively (16). Additionally, 3,19-analogue of 12-thioether andrographolide (Fig. 2C) has a cytotoxic effect on MCF-7 human breast cancer cell lines (17). The anticancer effects of andrographolide are also demonstrated in relation to human epidermoid carcinoma, breast cancer and human ovarian cancer cells via the nuclear factor-κB (NF-κB) signaling pathway. The results of the study demonstrate that andrographolide effectively inhibits the proliferation and growth of these cancer cell lines via the inhibition of this pathway (18). The chemical structures of andrographolide (19) and its derivatives are shown in Fig. 2.

Andrographolide has been tested with severe acute respiratory syndrome (SARS)-coronavirus (CoV)-2 to determine its ability to inhibit SARS-CoV-2 activity in human lung epithelial cells, Calu-3. Andrographolide inhibits the production of SARS-CoV-2 virions, with an IC_50_ of 0.034 µM. The results reveal that the anti-viral mechanism of action of andrographolide on SARS-CoV-2 is to target the non-structural proteins of the virions (20). Andrographolide also exhibits a bacteriostatic antibacterial action against bacteria (21).

Furthermore, an *in vivo* analysis using *Plasmodium berghei* and three different andrographolide fractions in tablet form reveals that the inhibition rate of the growth of this parasite ranges between 70-80%, demonstrating the anti-parasitic effect of andrographolide (22). Another potent pharmacological effect of andrographolide is its anti-inflammatory property. Andrographolide can suppress the inflammatory mediators in ovalbumin-stimulated mice by inhibiting the activation of the Janus kinase 1 (JAK1)/signal transducer and activator of transcription 3 (STAT3) signaling pathway by attenuating the production of TH17-regulated cytokines. This further demonstrates that andrographolide can treat inflammation-mediated diseases (23). In addition, andrographolide also has an antidiabetic effect, which is indicated in an *in vitro* study conducted on 3T3-LI mouse adipocytes. Andrographolide increases the glucose uptake by increasing the main components of glucose homeostasis, such as peroxisome proliferator-activated receptor γ and glucose transporter type 4(24).

Of these activities, the anticancer properties of andrographolide have received the most attention, as reflected by the number of studies investigating its potential in combating cancer (16-18). Previous reviews have addressed the anticancer effects of andrographolide but have not investigated the associated biochemical signaling pathways. Thus, the present review focused on the anticancer effects of andrographolide, specifically on breast, colorectal and lung cancer through the NF-κB, hypoxia-inducible factor 1 (HIF-1) and the JAK/STAT signaling pathways. Therefore, the Google Scholar, PubMed and ScienceDirect databases were used to search for references to these prevalent cancers and the anticancer mechanisms of andrographolide. The following key words were used: Andrographolide, anticancer, NF-κB, HIF-1, JAK/STAT, phosphatidylinositol-3-kinase (PI3K)/AKT/mammalian target of rapamycin (mTOR), Wnt/β-catenin and mitogen-activated protein kinase (MAPK) pathways, and the literature was limited to studies published between 2010 to 2023. The present review article detailed the different involvements of the signaling pathways in the anticancer mechanisms of andrographolide.

## 2. Types of cancer and current treatments

The anticancer effects of andrographolide and its derivatives have been gaining considerable attention among researchers due to its low toxicity towards the human body (13). The present section introduces three different types of cancer: Breast, lung and colorectal cancer.

### Breast cancer

According to data collected by the World Health Organization in 2020, breast cancer is a prevalent form of cancer (surpassing lung cancer), with 2.26 million newly diagnosed cases recorded worldwide in 2020 alone (25). Breast cancer is recognized as one of the most prevalent types of cancer to affect women worldwide. However, the most severe type of breast cancer is triple-negative breast cancer (TNBC) which has notable long-term adverse effects as it is more aggressive compared with the other types (25). Only a small number of effective targeted treatments are available for this type of breast cancer, hence the importance to investigate the causes and effects of breast cancer in order to address the current need for its effective treatment. Currently, the available treatment to stop metastasis of TNBC cells and early-stage TNBC is cytotoxic chemotherapy (26,27), which causes an array of side effects such as hair loss, peripheral neuropathy, anemia and fatigue (28). However, several costly drugs have been approved by the United States Food and Drug Administration, such as capecitabine, bevacizumab, albumin bound-paclitaxel and docetaxel (29). Traditional Chinese medicine (TCM) is another option that patients may resort to when modern medicine fails. TCM for breast cancer prevention often involves acupuncture and herbal medicine, which are used 19.4 and 100% of the time in TCM, respectively (30,31). The numerous herbal agents often used to prevent breast cancer include *Astagaslia macrocephalia*, *Salvia miltorrhizae*, *Atractlodes* and *Poria cocos* (31). Studies on patients with chronic fibrocystic breast disorder treated with TCM suggest that this approach is effective and does not have adverse side effects (31,32). Natural components can also be useful in cancer therapy and prevention. Studies demonstrate that andrographolide can suppress levels of different factors, such as tumor necrosis factor-α (TNF-α), monocyte chemoattractant protein-1, high sensitivity c-reactive protein and interleukin (IL)-1β. Therefore, andrographolide is recognized as an anti-inflammatory agent in macrophages for different types of diseases such as breast cancer, hepatitis, coronary heart and pulmonary fibrosis (33-35).

Women have a higher incidence of developing breast cancer compared with men, as the lifetime risk of developing breast cancer for a man is 1:100 compared with 1:8 for a woman. In men, the rate of developing all types of breast cancer, primarily estrogen-receptor-positive, is only ~1% (36). Therefore, it is important to understand how tumor progression and metastasis work. The tumor microenvironment, which refers to the ecosystem surrounding a tumor in the body, can influence tumor progression and metastasis. In a tumor microenvironment, elements that serve a major role in tumor development are angiogenesis, fibrosis, immune cells and macrophages (25). However, that tumor-associated macrophages (TAM) have the greatest influence on breast cancer development as they comprise 5-40% of the mass of solid tumors (37,38). The two main subtypes of TAM are classically activated macrophages, M1, and alternatively activated macrophages, M2. They have contrasting roles in TAM, where M1 macrophages are activated by the microbial product interferon-γ (IFN-γ), while M2 macrophages are induced by Type 2 helper T-cell cytokines. M1 are known as proinflammatory and cancer-cell-killing macrophages, while M2 are anti-inflammatory macrophages that aid in tissue repair processes and the expression of scavenger receptors, such as CD163, CD23 and CD206, to initiate various signaling pathways, such as the NF-κB and PI3K/AKT/mTOR pathways, in the tumor microenvironment, including those that result in tumor progression (38,39). Consequently, agents that re-polarize macrophages from a pro-inflammatory to an anti-inflammatory subset or regulate secreted cytokines of the M1 subtype are useful tools for cancer therapy (38,39).

### Colorectal cancer

With a malignancy rate that is ranked as second worldwide, colorectal cancer has a high mortality rate of 35%, and 147,950 individuals were diagnosed with this type of cancer in 2020 alone (40). Patients were mainly >50 years old, with a mortality rate of 20% (41). This complex multifactorial disease involves genetic mutations and epigenetic mutations, resulting in gene changes associated with processes such as cellular differentiation and colonocyte regulation (42). In the initial phase, the cancer cells develop from the colon mucosa crypt, which is a tube-shaped gland in the colon wall, and start to proliferate. In the first stage, the cancer cells originating from the mucosa wall start to emerge onto the muscularis propria and submucosa. At this point, patients have >90% chance of surviving 5 years. In the second stage, which has a 50% survival rate, cancer cells continue to develop into the attached organs, visceral peritoneum and peri-colorectal tissues. In the third stage, the cells further metastasize to nearby tissues and lymph nodes (43,44). The cancer cells then metastasize to other organs such as the lungs, liver or ovaries, indicating the start of stage four of colorectal cancer, for which the survival rate is only 10% (43,44). The high invasiveness and metastatic behavior of colon cancer cells mean they surpass lung cancer in terms of the mortality rate. The nature of this disease makes it challenging to detect until it reaches an advanced stage (45).

Several treatments are now available, such as chemotherapy, surgery and targeted therapy. However, these treatments are not promising because chemotherapeutic drugs can cause adverse toxicity and drug resistance could potentially develop over time. In addition, targeted therapy demonstrates a lack of cost-effectiveness and specificity, and it has the potential to be associated with adverse events such as leukopenia or hypertension (45). Common systemic therapy consists of chemotherapy with the drug fluoropyrimidine, taken in isolation. However, treatment might also involve other types of drugs, such as oxaliplatin or irinotecan, as well as biological therapy that targets vascular endothelial growth factor (VEGF), multiple receptor tyrosine kinases or epidermal growth factor receptors (46,47). TCM can also be applied in treating colorectal cancer as it helps to induce apoptosis and cycle arrest, suppress migration and invasion, and target the tumor microenvironment. TCM compounds associated with anti-colorectal cancer agents are berberine, evodiamine, shikonin, quercetin, resveratrol and curcumin (46). Berberine is a traditional herbal medicine obtained from the plant *Coptis chinensis* (48). It downregulated 33 genes differently involved in the cell cycle, epithelial-mesenchymal transition (EMT) and differentiation when tested on HCA-7 cell lines with doses of between 10 and 100 µM (49). Phytochemicals are natural products that can inhibit the activities of cancer cells, such as cell proliferation, angiogenesis and inflammation, but have minimal side effects (50). Andrographolide, a phytochemical with anticancer properties, has been used worldwide as a cancer treatment. This compound acts on regulatory pathways such as the cell adhesion process, the cell cycle and apoptosis (51). A previous study using human colorectal carcinoma LoVo cells treated with andrographolide reveals an anti-proliferative effect on the cells in a time- and dose-dependent manner. Even when treatment involves a low concentration of andrographolide, such as 0-30 µM, andrographolide still causes cell arrest at the G1-S phase, an interphase stage in the cell cycle (52). Additionally, andrographolide induces protein expression in p53, p21 and p16, resulting in suppression of cyclin D1/Cdk4, cyclin A/Cdk2 and the Rb phosphorylation process. Thus, andrographolide can inhibit the proliferation of LoVo cells (52).

### Lung cancer

According to the Global Cancer Observatory, the global mortality rate of lung cancer caused by neoplasms of the lungs was 1.8 million, with 2.2 million cases of lung cancer reported in 2020 alone, making it the most common cancer-related cause of mortality worldwide (25). The two types of lung cancer are small-cell lung cancer (SCLC) and non-small lung cancer (NSCLC). Of these, SCLC is known to be the more malignant, and it comes from cells with neuroendocrine features. Most cases of lung cancer originate from NSCLC, accounting for 85% of cases, compared with SCLC that accounts for 15% of cases. Furthermore, NSCLC can be divided into three pathologic subtypes: Large cell carcinoma, squamous cell carcinoma and adenocarcinoma (53). The high number of NSCLC cases is due to poor diagnosis and tumors worsening over time (53).

There are several uncontrollable risk factors for lung cancer, such as age, sex, ethnicity and family history. In the United States, the average age for lung cancer diagnosis is 70 years old, with >50% of cases being in the age range of 55-74 years (54,55). This is because mutagen exposure occurs over a long period of time often spanning several years or even decades before causing carcinogenesis, which delays the detection of the illness. Furthermore, as individuals age, the telomeres shorten, leading to a decrease in the metabolite NAD+ and cells no longer being able to repair and withstand DNA damage or detect abnormal cells (56). In terms of sex differences, men are more likely to be diagnosed with and die from lung cancer, with the lifetime risk of diagnosis being 3.80% among men and 1.77% among women. This increased risk among men is attributed to a higher frequency of tobacco smoking in men, which is 36.7% globally compared with 7.8% for women (55,57). Additionally, one theory suggests that hormones might influence the relatively low occurrence of lung cancer in women. This hypothesis stems from the overexpression of estrogen receptors in adenocarcinomas and the antitumor effects of antiestrogen compounds observed in *in vitro* studies (55,58). Ethnicity also serves a major role in the number of lung cancer cases. Despite having lower rates of cigarette consumption (~2%), Chinese women are equally as likely to be diagnosed with lung cancer as Western European women, who have a smoking prevalence of 20-25%. This is due to air pollution and charcoal burning, which exposes Chinese women to smoke more frequently compared with Western European women (59). However, patients with lung cancer may have no history of smoking tobacco, suggesting that the disease could be due to heritable components (55). According to studies by the Genome-wide Association, certain variants in several chromosomal regions, such as the 5p15 locus (60), 6p21 locus (61) and 15q25-26 loci, might increase the probability of being diagnosed with lung cancer (55,62).

The current available treatments for lung cancer are targeted therapy, chemotherapy, radiotherapy and surgical resection. Patients with stages I and II of NSCLC are suggested to undergo surgical tumor resection as this is the most effective therapy, but it has limited effects among patients in the advanced stages (53). The two types of chemotherapy are platinum-based chemotherapy for NSCLC (63) and standard chemotherapy for SCLC (64). Targeted therapy and platinum-based chemotherapy have demonstrated a number of therapeutic benefits (65). However, these treatments are also associated with a number of systemic toxicities and adverse effects such as diarrhea, vomiting, fatigue and neutropenia (66,67). The novel drugs resulting from pharmaceutical developments are known to have less severe side effects, unlike other common cytotoxic drugs. The available drugs include sorafenib, sunitinib and ASA404(68). TCM is also applied in lung cancer treatment paired with chemotherapy. The most popular therapy is a combination of TCM and platinum-based chemotherapy as this offers synergistic therapeutic effects and is reported to decrease severe toxicities by 36%. The TCM injection is called an Aidi injection, which is a common adjuvant chemotherapy drug used in China (69). Natural compounds such as andrographolide have good inhibitory effects on pro-survival autophagic processes. These processes involve the suppression of the PI3K/AKT signaling pathway, leading to a decrease in the primary factor responsible for tumor growth in NSCLC cells, which is HIF-1α (70,71). A recent study on NSCLC cell lines, specifically H460 and H1650, treated with andrographolide demonstrates that the compound is able to induce ferroptosis, an iron-dependent cell death, which inhibits cell line growth and metastasis in H460 and H1650. The induction of ferroptosis was confirmed by the high levels of glutathione (GSH), malondialdehyde, reactive oxygen species (ROS), intracellular iron content and lipid ROS-reduced GSH. This further demonstrates that andrographolide can suppress NSCLC cell proliferation and metastasis (72).

### Other types of cancer

One of the leading causes of cancer-related mortality is liver cancer. This starts in hepatocytes, cells in the liver tissue that have a number of key functions, such as detoxification, clotting and lipid metabolism. Hepatocellular carcinoma is the most common type of primary liver cancer, comprising 75-85% of all liver cancer cases, and starts and progresses in hepatocytes (73). In the early stage of hepatocellular carcinoma, patients are asymptomatic but start to show symptoms such as fatigue and liver dysfunction as the cancer advances (73). The current treatment for this disease can involve surgical resection, liver transplant, ablation or embolization but these have numerous potential side effects such as a risk of infection, fever, nausea, damage to healthy liver tissue, bleeding and low survival rates, depending on the cancer stage (74). Drugs given to patients with hepatocellular carcinoma vary depending on the severity and progress of the cancer. Currently, sorafenib is given to patients as the first-line treatment, followed by negorafenib as the second-line treatment. These drugs help prevent cancer cells from multiplying and inhibit the tumor growth-promoting pathway (75). TCM may also be used to treat hepatocellular carcinoma, with herbal remedies, acupuncture, dietary therapy, exercise and massages being utilized in hospitals in China. Various Chinese herbal medicines are recognized by the International Organization for Standardization and may be prescribed to treat patients with hepatocellular carcinoma, such as *Poria (Fuling)*, *Rhizoma Atractylodis Macrocephalae* and *Radix Astragali Mongolici* (76). These herbs inhibit proliferation and tumor growth while reducing the side effects of common liver cancer treatments (76). Recently, andrographolide was investigated for its ability to treat liver cancer. Andrographolide coupled to phytosomes, which enhances the solubility and delivery of the compound to HepG2 liver cancer cells, demonstrates an ability to inhibit HepG2 cell proliferation. These findings indicate that andrographolide coupled to phytosomes notably suppresses HepG2 cell proliferation, with an IC_50_ value of 4.02±0.14 µM (73).

In 2024, it is predicted that there will be 2 million new cancer cases in the United States, with an estimated 299,010 cases of prostate cancer. Additionally, a high number of mortalities, totaling 35,250 cases, are expected in the United States due to this cancer (77). In the United States, prostate cancer is the leading cancer-related cause of mortality among the male population (78). The currently available first-line treatments of prostate cancer are surgery, radiation therapy and proton beam therapy. Additionally, chemotherapy, hormonal therapy, cryosurgery and high intensity focused ultrasound are also conducted to treat patients with prostate cancer, depending on the severity and stage of the cancer. However, these treatments expose patients to a number of side effects that may hinder aspects of their quality of life such as urinary issues and erectile dysfunction (79). Several drugs categorized as second-generation non-steroidal anti-androgens are currently used as a pharmacotherapy for patients with prostate cancer, namely enzalutamide, apalutamide and darolutamide. These drugs compete against testosterone and dihydrotestosterone to bind to the androgenic receptor, inhibiting cell proliferation and the tumor growth of prostate cancer cells. However, a number of these drugs may cause side effects such as hypertension, bone injury and diarrhea (80). TCM has also been used among patients with prostate cancer as an alternative treatment. A study on the Chinese medicine extract *Ganoderma lucidum* demonstrates its ability to induce apoptosis, inhibit cell proliferation and suppress PC3 human prostate cancer cells from metastasizing to other organs, with a concentration of 0.125-0.5 mg/ml (81). A previous study investigated the ability of andrographolide to inhibit prostate cancer cell activity. It reveals that andrographolide can suppress tumor growth in mice induced with a castration-resistant DU145 human prostate tumor by inhibiting the protein expression of IL-6. This was indicated by a 50% reduction in the IL-6 protein in DU145 cells after treatment with 3 µM andrographolide (82). Similar results are observed in the human prostate cancer cell line PC3(82).

Leukemia is a type of cancer that originates in the bone marrow and causes the production of large numbers of abnormal white blood cells. The four types of leukemia are: Acute lymphoblastic leukemia (ALL), acute myelogenous leukemia, chronic lymphocytic leukemia and chronic myelogenous leukemia (83). Of these, ALL is the most aggressive and is known for its ability to metastasize to other organs. The current treatments available for leukemia are induction chemotherapy and risk-directed therapy. The current survival rate of young patients aged 1-9 years with ALL is 80-90% due to implementing newly developed chemotherapy schedules and optimized risk-directed therapy (84,85). However, studies have reported that cell resistance and relapse are still the greatest challenge in treating ALL (84,86,87). Drugs have also been used with chemotherapy; a method known as combination therapy. Common drugs used in this way are glucocorticoids (GCs) and dexamethasone. GCs aid in regulating genes that cause apoptosis in leukemia cells by activating the glucocorticoids receptor; however, long-term exposure to GCs may cause adverse reactions, resulting in resistance to this drug (84). TCM herbs are used to treat young patients with leukemia, including treatments based on *Radix Astragali membranaceus*, *Bulbus Fritillariae thunbergii* and *Herba Hedyotisdiffusa*. Furthermore, three common types of TCM used to treat adult patients (aged 19-80 years old) with leukemia are *Radix Astragali membranaceus*, *Radix et Rhizoma Salviae miltiorrhizae* and *Fructus Ligustri Lucidi.* These herbs are known for their ability to increase the white blood cell count of a patient (88). The anti-tumor activity of andrographolide toward human T-ALL Jurkat cells was investigated using both *in vitro* and *in vivo* (nude mice models) analysis. The results demonstrate the ability of andrographolide to induce apoptosis in Jurkat cells, with a concentration of 10 µg/ml. Additionally, the *in vivo* study on T-ALL Jurkat cells with three different concentrations of andrographolide (50, 100 and 200 mg/kg), demonstrates that the compound notably inhibits the growth of T-ALL Jurkat cells in mice (89). This further demonstrates the anti-tumor activity of andrographolide in leukemia.

## 3. Mechanisms of andrographolide activation and its derivatives

Andrographolide, known for its function as a natural antibiotic, has various other abilities and can act to reduce fever, remove toxins, relieve pain and reduce inflammation (90). A number of pathways allow andrographolide to exert its anticancer effects; however, the main signaling pathways are the NF-κB, HIF-1 and JAK/STAT signaling pathways. Other pathways have also been discussed in the present review, such as the PI3K/AKT, mTOR, Wnt/β-catenin and MAPK signaling pathways (91). Table I summarizes the anticancer effects of andrographolide through these pathways.

### Inhibition of the NF-κB signaling pathway

**Figure 3:**
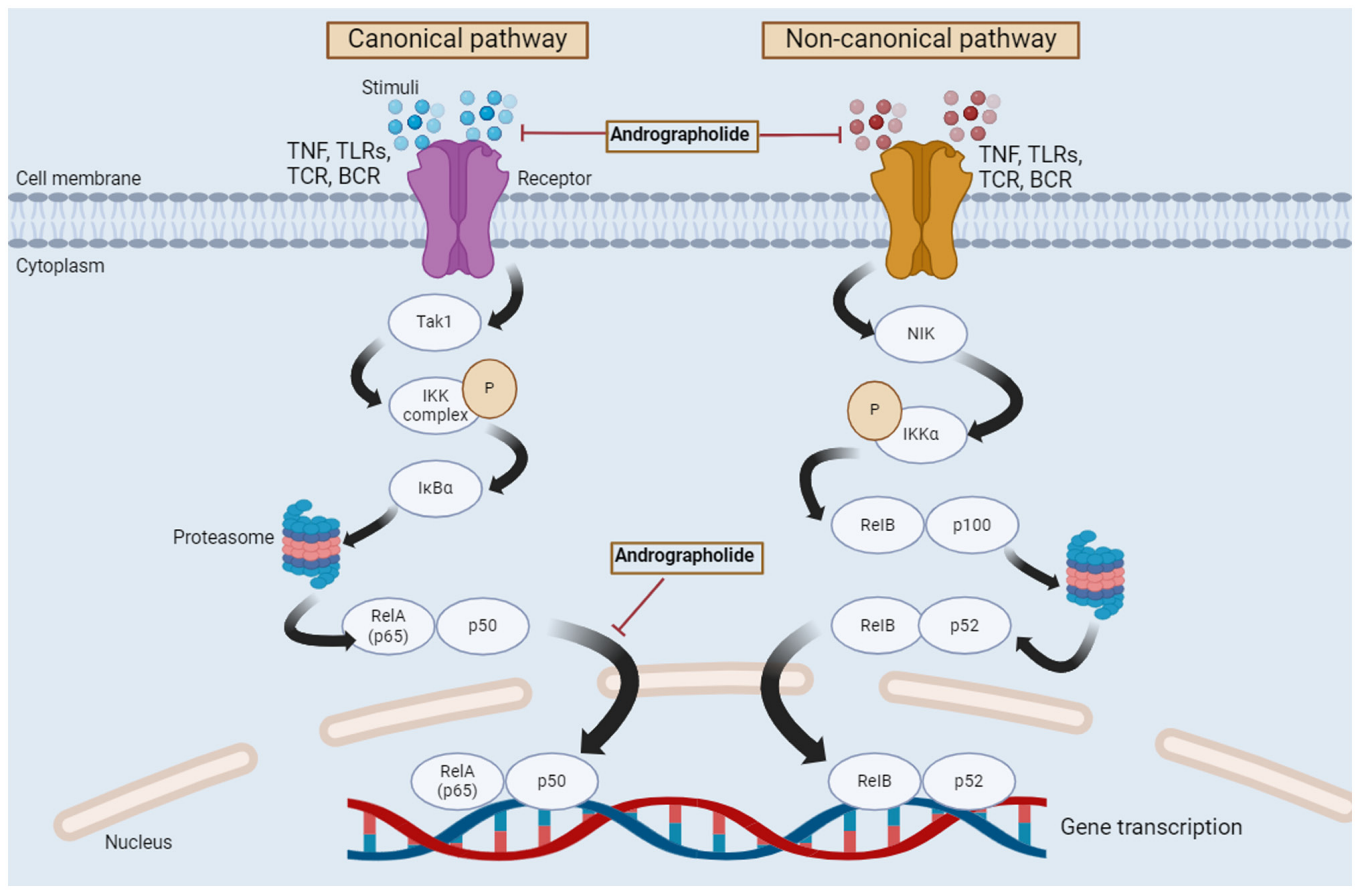
Regulation of canonical and non-canonical NF-κB signaling pathways by andrographolide. Two different pathways, namely the canonical and non-canonical pathways, form the NF-κB signaling pathway. The canonical pathway begins at the cell membrane, where different stimuli bind to their corresponding receptors. This causes the activation of the IKK complex by Tak1. Subsequently, IκBα is phosphorylated in an IKK-mediated manner, and the phosphorylated IκBα is degraded by the proteasome. The degraded and phosphorylated IκBα then forms a heterodimer, RelA/p50, which leads to the nuclear translocation of this heterodimer and results in gene transcription. The non-canonical pathway begins with stimuli binding to a restricted set of cell surface receptors, namely TNF and B cell activating factor receptors, leading to the activation of IKKα by NIK. Subsequently, IKKα is phosphorylated and the RelB/p100 complex undergoes a partial proteolysis by the proteasome, which activates p52. RelB dimerizes with p52 and forms a heterodimer, which then translocates to the nucleus and causes gene transcription. Tak1, TGF-β activated kinase-1; IKK, IκB kinase; NF-κB, nuclear factor-κB; NIK, NF-κB inducing kinase; RelA, REL-associated protein; RelB, RELB-associated protein; p65, REL-associated protein; TNF, tumor necrosis factor; TLRs, toll-like receptors; TCR, T-cell receptor; BCR, B-cell receptor; IκBα, inhibitory κ B α; P, phosphorylation.

NF-κB is a signaling pathway that regulates various cellular processes such as the production of anti-tumor cytokines, gene transcription and the inhibition of apoptosis (92,93). Aside from the ability of anti-tumor cytokines to combat cancer, they can also manipulate signaling pathways (such as the NF-κB, PI3K/Akt and JAK/STAT pathways) to avoid immune detection and destruction (94-96). In response to chemotherapy, cancer cells can inhibit apoptosis and produce anti-tumor cytokines in order to evade the immune system through different processes such as chronic inflammation, over expression of anti-apoptotic proteins and a modulation of the immune response (97). This immunoevasive strategy allows cancer cells to survive and proliferate despite the immune system (92). The NF-κB pathway is one of the transcription factors that serves a major role in a number of physiological and pathological processes. Physiological processes include immunological responses, inflammation and apoptosis, whereas pathological processes include cancer, autoimmune diseases and metabolic disorders (98,99). The pathway is divided into two different pathways, canonical and non-canonical, with each having different activating mechanisms (98,100). Activation of the canonical NF-κB pathway causes a number of different responses, which are involved in survival, differentiation, cell proliferation, immune response and inflammation (98,101). The non-canonical NF-κB pathway is mainly associated with the development of immune cells in various stages, and it is a critical regulator in the development of secondary lymphoid organs (98,100), the development of tertiary lymphoid organs and chronic inflammation (102). For immune cells that are associated with the development of T cells in the thymus, this pathway is needed for thymus epithelium cell maturation and function (98,103). The NF-κB canonical pathway begins at the cell membrane, where different stimuli (such as carcinogens, tumor promoters, stress, endotoxin, apoptosis-inducers, infection, reactive oxygen intermediate inducers and cytokines (104) bind to their corresponding receptors, such as the TNF, toll-like receptors and T/B cell receptors (105). This causes the activation of the IκB kinase (IKK) complex by TGF-β activated kinase-1, which takes place in the cytoplasm. Subsequently, IκBα is phosphorylated in an IKK-mediated manner, and the phosphorylated IκBα is degraded by the proteasome. Thus, the degraded phosphorylated IκBα forms a heterodimer, RelA/p50, which leads to the nuclear translocation of this heterodimer and results in gene transcription. In contrast, the non-canonical pathway begins with stimuli binding to a restricted set of cell surface receptors, namely TNF and B cell activating factor receptors, leading to the activation of IKKα by NF-κB inducing kinase. Subsequently, IKKα is phosphorylated and the RelB/p100 complex undergoes partial proteolysis by proteasome-activating p52. RelB dimerizes with p52 and forms a heterodimer, which translocates to the nucleus and causes gene transcription (106-109). Fig. 3 indicates the regulation of the NF-κB signaling pathway by andrographolide.

NF-κB is a gene transcription factor that serves an important role in immune responses and cellular inflammation. Due to its pro-inflammatory function, the NF-κB signaling pathway may cause several autoimmune diseases including cancer, as well as aiding the survival, proliferation, metastasis, angiogenesis and further development of cancer cells (110). Therefore, the NF-κB signaling pathway has gained considerable attention, especially in terms of developing drugs that can inhibit this signaling pathway to overcome the different types of cancer to which it is associated with (111). Different stimuli that bind to different receptors may activate the transcription and expression of various genes, such as, invasion-related genes and anti-apoptotic genes. This process also regulates cell proliferation and angiogenesis. The gene transcription process induces matrix metalloproteinases (MMPs), which are invasion-related genes that promote cancer cell invasion, adhesion and metastasis (109) MMP-9 is an MMP protein that specifically causes breast cancer cells to metastasize to other organs, while MMP-11 is known to cause the proliferation of prostate cancer cells (112,113). Therefore, the downregulation of these MMP proteins using andrographolide could prevent these cancer cells from metastasizing and proliferating by inhibiting the phosphorylation process, which in turn, halts the gene transcription process (109,114). TNF-α and IL-8 are cytokines that bind to the TNF and CXC chemokine receptors, respectively to initiate the NF-κB signaling pathway, which causes the angiogenesis of colorectal cancer (115-117). Andrographolide downregulates inflammatory factors such as TNF-α and IL-8, and thus, inhibits cytokine binding, resulting in an inhibition of angiogenesis-associated gene transcription (114). In a lipopolysaccharide (LPS)-induced RAW 264.7 cell line (originating from an Abelson leukemia virus-transformed cell line derived from BALB/C mice), andrographolide suppresses the activation of the NF-κB pathway, and thus hinders the inflammation induced by LPS. This also occurs due to the inhibition of the release of cytokines that activate the NF-κB pathway, such as TNF-α, IL-6 and IL-1β, when treated with doses of andrographolide ranging from 6.25 to 25.00 g/ml. The expression of these cytokines decreases as the andrographolide dose increases from 6.25-25.00 g/ml. Furthermore, the expression of the transcription factor p65 protein notably reduced, leading to the cessation of the gene transcription process in the nucleus (118). Another study using the human colon cancer SW620 cell line treated with 20 µM of andrographolide, reveals a notable reduction in the p65 protein expression levels, which are involved in the NF-κB signaling pathway, leading to the inactivation of the pathway (112). Additionally, a previous study using two different breast cancer cell lines of spontaneous luminal-like breast cancer, MMTV-PyMT and MCF-7, demonstrates a reduction in tumor growth after andrographolide treatment is applied. The high expression of p65 and pp65 (Ser536) proteins in breast cancer cell lines is reduced by andrographolide, which helps to prevent gene expression processes in the nucleus during the NF-κB signaling pathway (119). Furthermore, another study on pelvic inflammatory disease also indicates the ability of andrographolide to block the pathogen-induced activation of the NF-κB pathway by reducing the excessive production of chemokines and cytokines such as IL-1β, IL-6, C-X-C motif chemokine ligand 1, monocyte chemoattractant protein-1 and regulated on activation, normal T cell expressed and secreted (120). A notable decrease in pp65 levels is observed in the epidermoid carcinoma cell line, A431, and the breast cancer cell line, MDA-MB-231, as the andrographolide concentration increases from 10-50 µM. This prevents the growth of cancer cell and the proliferation process by inhibiting the NF-κB signaling pathway (18). Uncontrolled DNA damage in response to genotoxic stress, such as radiation or chemotherapy, will provide a challenge to cell homeostasis because it can disrupt genomic stability and survival, leading to cancer cell formation. DNA damage is able to initiate both the canonical and non-canonical NF-κB signaling pathways (121). A previous study using osteosarcoma cell lines demonstrates the ability of DNA damage to initiate the NF-κB signaling pathway following the phosphorylation of p100 and processing of the p52 protein through a non-canonical pathway (121). However, it remains unclear how DNA damage can activate the NF-κB signaling pathway through a non-canonical pathway and the role of IKKα in this pathway. Further research into the non-canonical NF-κB signaling pathway triggered by DNA damage may aid the prevention of cancer cell development (121).

### Inhibition of the HIF-1 signaling pathway

**Figure 4:**
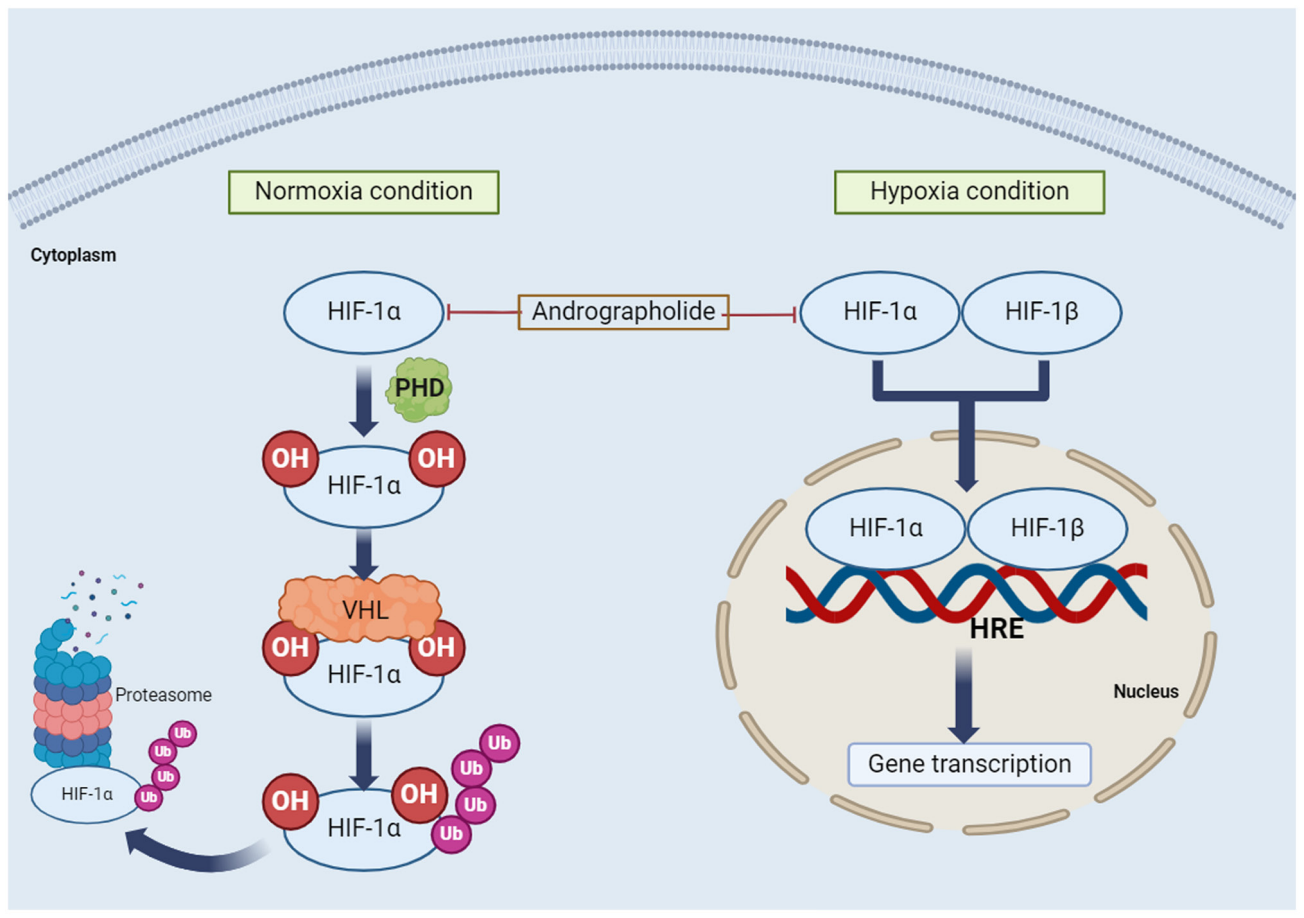
Regulation of HIF signaling pathways by andrographolide. Two conditions are associated with the HIF-1 signaling pathway: Normoxia and hypoxia. Under normoxic conditions, proline residues on HIF-1α are hydroxylated by the PHD protein, forming a binding site for the VHL protein. Subsequently, Ub of HIF-1α is followed by degradation of polyubiquitylated HIF-1α in the 26S proteasome. Under hypoxic conditions, HIF-1α and HIF-1β form a heterodimer that binds to the HRE on DNA, activating hypoxia-responsive gene transcription. HIF, hypoxia-inducible factor; PHD, prolyl hydroxylase domain; VHL, von Hippel-Lindau; HRE, hypoxia responsive element; OH, hydroxylation; Ub, ubiquitylation.

HIF is a transcription factor, which activates genes that regulate cellular oxygen homeostasis, participate in physiological processes and react to environmental stressors. Physiological processes include angiogenesis and cell proliferation, whereas environmental stressors include hypoxia and inflammation (122). The deregulation of HIF expression is involved in cancer progression and metastasis. The HIF-1 signaling pathway is activated in cancer cells through growth factors such as TGF-β3 and epidermal growth factors (123). Two conditions are associated with the HIF-1 signaling pathway: Normoxia and hypoxia. Under normoxic conditions, proline residues on HIF-1α are hydroxylated by the prolyl hydroxylase domain protein, forming a binding site for the von Hippel-Lindau protein. Subsequently, ubiquitylation (Ub) of HIF-1α is followed by degradation of polyubiquitylated HIF-1α in the 26S proteasome (124,125). Under hypoxic conditions, HIF-1α is not hydroxylated, but HIF-1α and HIF-1β form a heterodimer (124,125) that binds to the hypoxia-responsive element on DNA, activating hypoxia-responsive gene transcription. This results in the expression of genes such as VEGF, erythropoietin and adrenomedullin, which cause angiogenesis, erythropoiesis and cell survival (123). The regulation of the HIF signaling pathway by andrographolide is presented in Fig. 4.

Metastasis and tumorigenesis can be promoted by altering the tumor microenvironment through inflammation and hypoxia (126). Tumorigenesis causes vessels to be leaky and abnormal, resulting in enlarged hypoxic regions. This promotes metastasis and makes the tumor resilient to current treatments such as radiotherapy and chemotherapy (126,127). HIF activity in immune cells, HIF pathway stabilization and HIF expression can be triggered by hypoxia and pathological stress such as cancer or inflammation (126). Inhibition of the HIF-1 signaling pathway by andrographolide occurs in MDA-MB-231 and T47D breast cancer cells under hypoxic conditions through its targeting of the expression of HIF-1α mRNA and HIF-1α protein levels via processes such as protein translation or degradation, which terminates the proliferation process of these breast cancer cells (123,126,128). Additionally, in Hep3B liver cancer cells, andrographolide can reduce HIF-1α protein expression, reducing its nuclear translocation. Andrographolide also downregulates a pro-angiogenic growth factor, VEGFA, which activates the HIF-1 signaling pathway in Hep3B cancer cells (129,130). Furthermore, HIF-1 is responsible for lung cancer growth in A549 cells and NSCLC. A previous study reveals that andrographolide inhibits the HIF-1 signaling pathway in A549 cells by reducing VEGF, leading to the inactivation of the HIF-1 signaling pathway (71). Thus, andrographolide serves a role as an anti-angiogenesis and is potentially a chemotherapeutic drug for treating NSCLC (14). Histone deacetylase (HDAC) is a redox-sensing deacetylase that can oppose the target gene expression of HIF-1α, but during hypoxia, it may cause activation of the HIF-1α pathway. It remains unclear how HDAC inhibitors can inhibit the HIF-1α signaling pathway (123). Therefore, in-depth research on the inhibition of HDAC by andrographolide will enhance the understanding of how it can inhibit this signaling pathway by disrupting HIF-1α stabilization.

### Inhibition of the JAK/STAT signaling pathway

**Figure 5:**
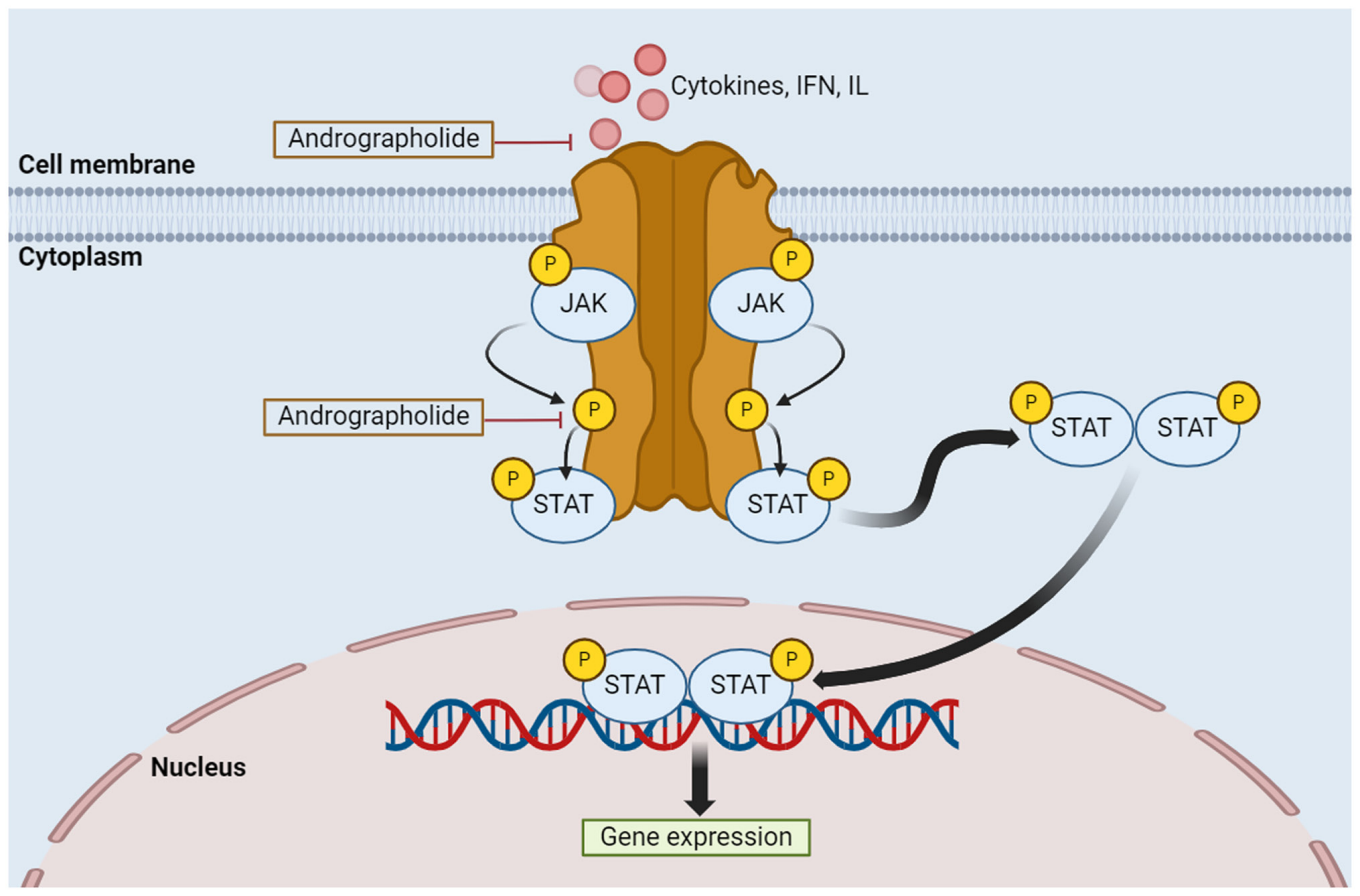
Regulation of the JAK/STAT signaling pathway by andrographolide. Activation of this process is the result of reactions outside of the cell, in which cytokines, IFN and IL, attach to their specific receptors, resulting in JAK proteins phosphorylating each other. JAK phosphorylates both the binding site of the STAT protein receptor and the STAT protein on its retained tyrosine residues, forming a complex STAT molecule in the cytoplasm. This molecule travels to the nucleus and attaches to the DNA, initiating the expression of genes. Types of receptors include growth factor receptors, IL receptors and G-protein-associated receptors. JAK, Janus kinase; STAT, signal transducer and activator of transcription; IFN, interferon; IL, interleukin; P, phosphorylation.

The JAK/STAT pathway is crucial for gene expression and controlling cell functions (131). However, abnormal activation, mainly due to the inappropriate stimulation or constitutive binding of a ligand to its receptor, can lead to tumorigenesis (132). The JAK/STAT signaling pathway mainly involves three proteins: Cell-surface, JAK and STAT receptors. The activation process begins with reactions outside of the cell, where cytokines, IFN and IL, attach to their specific receptors, resulting in JAK proteins phosphorylating each other. The receptors involved are growth factor, IL-specific and G-protein-associated receptors (82,131,133). JAK phosphorylates the STAT protein receptor allowing the STAT protein to bind before JAK then phosphorylates the STAT protein on its retained tyrosine residues, leading to the formation of a STAT protein complex in the cytoplasm. This molecule travels to the nucleus and is attached to the DNA, initiating the expression of genes that aid in tumor cell differentiation, proliferation and activation (133). Regulation of the JAK/STAT signaling pathway by andrographolide occurs as shown in Fig. 5.

The inhibition of the JAK/STAT pathway by andrographolide is further demonstrated in previous studies using different types of human cancer cells. IL is a type of cytokine that binds to a receptor on the cell membrane to initiate the JAK/STAT pathway. IL-6 is one of the cytokines produced by various types of lymphocytes as well as non-lymphocytes such as endothelial cells, monocytes, fibroblasts, and T and B lymphocytes (134). It facilitates the EMT process by activating the JAK/STAT pathway, which results in inflammation (134). Previous studies using DU145, AsPC-1 and Panc-1 cancer cells reveal that andrographolide can reduce the IL-6 expression levels and signaling, which is directly associated with the inhibition of the JAK/STAT pathway (82,135). Additionally, in the MDA-MB-231, AD-293, HI975 and HI299 cancer cell lines, andrographolide inhibits the phosphorylation of STAT3, hindering cell apoptosis, growth and proliferation (136-138). Hindering one of the steps in the JAK/STAT pathway may lead to cancer cell death (82). Other receptors that will be attached to the cell membrane include G-protein-coupled receptors and homodimeric hormone receptors with ligands such as bradykinin receptor B2 and thrombopoietin binding to them, respectively. The binding of these ligands to their receptors may cause ovarian and myeloproliferative cancer (139). Therefore, studies investigating how andrographolide suppresses these biomarkers will further the understanding of how it inhibits the JAK/STAT pathway and may elucidate its anticancer effects.

### Other signaling pathways

Andrographolide inhibits tumor growth in a number of other pathways. One of these is the PI3K/AKT/mTOR signaling pathway, which serves a major role in cancer cell growth and tumor proliferation by responding to different types of factors such as nutrients, hormones and growth factors. In this pathway, receptor tyrosine kinases bound to the cell membrane activate the PI3K molecule. This pathway begins with growth factors binding to ligand binding sites, causing the dimerization of the receptors, with one receptor phosphorylating a tyrosine residue on the other receptor. This allows the docking of proteins, such as insulin receptor substrate-1 and the GRB-2-associated binder, which are then activated by tyrosine kinases PI3K (140). The PI3K molecule phosphorylates a lipid bound to two phosphates, namely PiP2, to cause PiP3 activation, which further activates the AKT molecule. AKT will further initiate the downstream effects and activate mTOR. Subsequently, mTOR will upregulate cell translation to initiate the synthesis of proteins from mRNA as well as the biosynthesis of lipids. mTOR has roles in cell survival, cell cycle progression and cell division. The disturption or upregulation of mTOR pathways will lead to uncontrollable cell division, causing cancer (141). The Bcl-2 protein promotes cell survival when phosphorylated by AKT, unlike the Bax protein, which is a pro-apoptotic protein. The treatment of breast cancer cell lines, MCF-7 and MDA-MB-231, with andrographolide, demonstrates an inhibition of Bcl-2 and an increase in the expression and protein levels of Bax, further demonstrating the apoptotic effect of andrographolide on these breast cancer cell lines (142).

The Wnt/β-catenin pathway involves Wnt, a series of growth simulating factors, which is a protein with palmitoleic acid attached to it for binding purposes (143). β-catenin is a molecule mainly regulated through degradation. In an activated state, β-catenin is bound to a destruction complex, which is a large protein complex including axis inhibition protein, glycogen synthase kinase 3β, casein kinase I, adenomatous polyposis coli, dishevelled and β-transducin repeat-containing protein (144-146). The destruction complex is so named because it causes phosphorylation, Ub and proteasome degradation of β-catenin (147,148). This pathway begins with the activation of the Wnt receptors, such as Frizzled Class Receptor 1 and Frizzled Class Receptor 2, by Wnt, which leads to low-density lipoprotein receptor-related protein (LRP) phosphorylation. This induces the translocation of the destruction complex to the region of the membrane near the frizzled and LRP receptors (148,149). Subsequently, dishevelled binds to LRP and becomes activated, leading to destruction complex inhibition (150). This increases the levels of β-catenin in the cytosol because it does not undergo phosphorylation, Ub or degradation in the proteasome (150). The transcription factor (TCF) mediates gene expression as the β-catenin translocates into the mitochondria, dislodging Groucho from the TCF (150). Subsequently, β-catenin binds to the TCF, leading to the transcription of the Wnt target genes and the growth and proliferation of the cell (150-152). A study on the anticancer effect of andrographolide (using colorectal cancer lines, HCT116 and SW480, and both *in vitro* and *in vivo* analysis) demonstrates a dysregulation of the WNT16, transcription factor 7-like 2 and axis inhibition protein 2 protein expression levels in the cancer cells following treatment with andrographolide. The co-administration of andrographolide along with 5-fluorouracil-based chemotherapeutic regimens notably reduces the tumor volume in mice. These results further indicate the anticancer effect of andrographolide through the dysregulation of the Wnt/β-catenin pathway (153).

The MAPK pathway regulates processes such as cell death, cell differentiation and cell proliferation (154). An epidermal growth factor signaling molecule, located outside of the cell, binds to the epidermal growth factor receptor to activate the MAPK pathway. This process causes the dimerization and autophosphorylation of a tyrosine residue on the receptor, which leads to the recruitment of the growth factor receptor-bound protein 2 (GRB-2) protein, which contains the Src-homology 2 (sh2) domain (155,156). This protein is activated as it binds to the phosphorylated tyrosine through the sh2 domain, which then recruits another molecule, son of sevenless (SOS) protein, which is attached to the sh2 domain of GRB-2(157). Subsequently, the SOS protein activates the RAS molecule containing bound GDP by replacing it with GTP. Activated RAS phosphorylates the RAF molecule, which then activates the MEK molecule (156-158). The activated MEK molecule then targets and phosphorylates the ERK 1/2 molecule, which is the outcome of the kinase cascade. The ERK1/2 molecule acts on different transcription factors such as C-Fos and C-Jun, causing them to dimerize into activator protein-1 (AP-1) (156,157). AP-1 then enters the nucleus and binds to the DNA molecule, initiating gene transcription (156,157). Glioblastoma multiforme (GBM) is the most common type of primary malignant brain tumor in adults globally, accounting for 50.1% of all primary malignant brain tumors in the United States (with 14,490 new cases reported in the United States in 2023 alone), and it can be fatal due to its metastatic activity (159). An *in vitro* analysis using the brain tumor cell lines GBM8401 and U251, reports the prevention of GBM cell metastasis by andrographolide. The results reveal that andrographolide inhibits MMP-2 expression at the transcriptional level in the MAPK pathway (160). Since AP-1 is a key transcription factor that promotes MMP-2 transcription, decreasing the expression of MMP-2 facilitates the prevention of cancer progression and metastasis. Thus, it is crucial to target MMP-2 and the MAPK pathway to prevent tumor progression and enhance treatment efficiency.

## 4. Conclusion

In conclusion, the present review provides a comprehensive description of how andrographolide regulates the signaling pathways involved in the development of cancer cells, mainly NF-κB, HIF-1, JAK/STAT, PI3K/AKT/mTOR, Wnt/β-catenin and MAPK. Andrographolide has the potential to be an effective anticancer agent as it can target the important biomarkers involved in each of the signaling pathways. In the NF-κB signaling pathway, it mainly downregulates the MMP biomarkers. In addition, andrographolide specifically reduces the HIF-1α protein expression levels in the HIF-1 pathway, leading to unsuccessful gene transcription. In the JAK/STAT pathway, andrographolide targets both the IL-6 cytokine and the STAT3 protein, inhibiting the gene expression process, which can cause apoptosis as well as reduce cell growth and proliferation. Hence, andrographolide has been highlighted as a promising anticancer agent in various types of signaling pathways.

## 5. Future perspectives

The present review discusses the most prevalent types of cancer, such as lung, colorectal and breast cancer, as well as signaling pathways such as NF-κB, HIF-1, JAK/STAT, PI3K/AKT/mTOR, Wnt/β-catenin and MAPK. The present review also discusses the ability of andrographolide to inhibit these signaling pathways to prevent cancer cells from growing efficiently. However, numerous biomarkers are involved in each signaling pathway, and their association with andrographolide are yet to be revealed. In the HIF-1 pathway, HDAC may activate this pathway under hypoxic conditions. Therefore, inhibition of this HDAC biomarker will also inhibit this pathway. Further in-depth *in vivo* and *in vitro* analyses of how andrographolide could aid this inhibition process would help reveal its ability to inhibit the HIF-1 pathway (161). Furthermore, at present, only a small number of studies have investigated the ability of andrographolide to inhibit the JAK/STAT pathway by downregulating two specific biomarkers, bradykinin receptor B2 and thrombopoietin. These biomarkers are known to be associated with cancer cell growth in myeloproliferative and ovarian cancers. In addition, previous studies using osteosarcoma cell lines demonstrate the ability of DNA damage to initiate the NF-κB signaling pathway following phosphorylation of p100 and processing of p52 protein through a non-canonical pathway. However, it remains unclear how DNA damage can activate the NF-κB signaling pathway through a non-canonical pathway and the role of IKKα in this pathway. Further research into the non-canonical NF-κB signaling pathway triggered by DNA damage could aid the development of cancer cell treatments.

## Figures and Tables

**Table I:** Anticancer effects of andrographolide due to the inhibition of NF-κB, HIF-1, JAK/STAT, PI3K/AKT/mTOR, Wnt/β-catenin or MAPK signaling pathways.

Signaling pathway	Type of cancer	Process	Cancer cell lines	Mechanism of action	(Refs.)
NF-κB	Breast cancer	Gene expression	MMTV-PyMT and MCF-7	Downregulates the protein expression levels of p65 and pp65 (Ser536)	(119)
	Colorectal cancer	Proliferation	SW620	Downregulates the MMP-9 signaling pathway	(112)
	Prostate cancer	Proliferation	PC3 and 22RV1	Decreases the expression level of MMP-11	(78)
HIF-1	Lung cancer	Metastasis and invasion	A549	Decreases the HIF-1α cellular protein level	(71)
	Breast cancer	Angiogenesis and cancer cell growth	MDA-MB-231 and T47D	Reduces the protein and mRNA levels of HIF-1α	(128)
	Liver cancer	Cancer cell growth	Hep3B	Decreases the protein expression levels of HIF-1α	(130)
JAK/STAT	Prostate cancer	Apoptosis	DU145	Suppresses the expression of IL-6 as well as the signaling pathway	(82)
	Breast cancer	Apoptosis	MDA-MB-231	Reduces the STAT3 luciferase activity	(137)
	Liver cancer	Cancer cell growth	AD-293	Inhibits the phosphorylation and dimerization of STAT3 proteins	(138)
	Lung cancer	Proliferation	H1975 and H1299	Downregulates the phosphorylation of STAT3 proteins	(136)
	Pancreatic cancer	Proliferation	AsPC-1 and Panc-1	Suppresses the activation of IL-6-induced STAT3	(135)
PI3K/AKT/mTOR	Breast cancer	Apoptosis	MCF-7 and MDA-MB-231	Inhibits the expression of Bcl-2 and increases the expression of Bax	(142)
Wnt/β-catenin	Colorectal cancer	Proliferation	HCT116 and SW480	Dysregulates the WNT16, TCF7L2 and AXIN2 protein expression levels	(153)
MAPK	Glioblastoma multiforme	Metastasis	GBM8401 and U251	Inhibits the expression of MMP-2 at the transcriptional level	(160)

NF-κB, nuclear factor-κB; HIF-1, hypoxia-inducible factor 1; JAK, Janus kinase; STAT, signal transducer and activator of transcription; PI3K, phosphatidylinositol-3-kinase; mTOR, mammalian target of rapamycin; MAPK, mitogen-activated protein kinase; IL, interleukin; MMP, matrix metalloproteinases; mRNA, messenger RNA; p65, REL-associated protein; pp65 (Ser536), phosphorylated-p65 (Serine536); Bcl-2, B-cell lymphoma 2; WNT16, Wnt family member 16; AXIN2, axis inhibition protein 2; TCF7L2, transcription factor 7-like 2.

## Data Availability

Not applicable.
